# Crowd Sourcing a New Paradigm for Interactome Driven Drug Target Identification in *Mycobacterium tuberculosis*

**DOI:** 10.1371/journal.pone.0039808

**Published:** 2012-07-11

**Authors:** Rohit Vashisht, Anupam Kumar Mondal, Akanksha Jain, Anup Shah, Priti Vishnoi, Priyanka Priyadarshini, Kausik Bhattacharyya, Harsha Rohira, Ashwini G. Bhat, Anurag Passi, Keya Mukherjee, Kumari Sonal Choudhary, Vikas Kumar, Anshula Arora, Prabhakaran Munusamy, Ahalyaa Subramanian, Aparna Venkatachalam, Gayathri S, Sweety Raj, Vijaya Chitra, Kaveri Verma, Salman Zaheer, Balaganesh J, Malarvizhi Gurusamy, Mohammed Razeeth, Ilamathi Raja, Madhumohan Thandapani, Vishal Mevada, Raviraj Soni, Shruti Rana, Girish Muthagadhalli Ramanna, Swetha Raghavan, Sunil N. Subramanya, Trupti Kholia, Rajesh Patel, Varsha Bhavnani, Lakavath Chiranjeevi, Soumi Sengupta, Pankaj Kumar Singh, Naresh Atray, Swati Gandhi, Tiruvayipati Suma Avasthi, Shefin Nisthar, Meenakshi Anurag, Pratibha Sharma, Yasha Hasija, Debasis Dash, Arun Sharma, Vinod Scaria, Zakir Thomas, OSDD Consortium, Nagasuma Chandra, Samir K. Brahmachari, Anshu Bhardwaj

**Affiliations:** 1 Council of Scientific and Industrial Research (CSIR), Delhi, India; 2 Institute of Genomics and Integrative Biology, CSIR, Delhi, India; 3 Department of Biochemistry, Indian Institute of Science, Bangalore, Karnataka, India; 4 Acharya Narendra Dev College, University of Delhi, India; 5 Goethe University, Frankfurt, Germany; 6 PSG College of Technology, Peelamedu, Coimbatore, Tamil Nadu, India; 7 SASTRA University, Tirumalaisamudram, Thanjavur, Tamilnadu, India; 8 SDM College, Ujire, Karnataka, India; 9 Sree Narayan Guru College, Coimbatore, Tamil Nadu, India; 10 Maharshi Dayanand University, Rohtak, Haryana, India; 11 Amity Institute of Biotechnology, Amity University, Lucknow, Uttar Pradesh, India; 12 Bharathiar University, Coimbatore, Tamil Nadu, India; 13 Bharathidasan University, Palkalaiperur, Tiruchirappall, Tamil Nadu, India; 14 Bitvirtual patan Node, Hem. North Gujarat University, Patan, Gujarat, India; 15 Business Intelligence Technologies India Pvt Ltd., Bangalore, Karnataka, India; 16 Christ College, Vidya Niketan, Saurashtra University, Rajkot, Gujarat, India; 17 Department of Life Sciences, Hemchandracharya North Gujarat University, Patan, Gujarat, India; 18 Department of Biotechnology, University of Pune, Maharashtra State, India; 19 Indian Institute of Toxicology Research, CSIR, Lucknow, Uttar Pradesh, India; 20 Indian Statistical Institute, Kolkata, West Bengal, India; 21 Maulana Azad National Institute of Technology, Bhopal, Madhya Pradesh, India; 22 Shri Ram College of Pharmacy, Karnal, Haryana, India; 23 The Maharaj Sayajirao University of Baroda, Gujarat, India; 24 Pathogen Biology Laboratory, Department of Biotechnology, School of Life Sciences, University of Hyderabad, Hyderabad, Andhra Pradesh, India; 25 University of Kerala, Thiruvananthapuram, Kerala, India; 26 All India Institute of Medical Sciences, New Delhi, India; 27 Department of Biotechnology, Delhi Technological University, Shahbad Daulatpur, Delhi, India; 28 Bioinformatics Centre, Institute of Microbial Technology, CSIR, Chandigarh, India; 29 Faculty of Science, Institute of Biological Sciences, University of Malaya, Kuala Lumpur, Malaysia; Albert-Ludwigs-University, Germany

## Abstract

A decade since the availability of *Mycobacterium tuberculosis* (Mtb) genome sequence, no promising drug has seen the light of the day. This not only indicates the challenges in discovering new drugs but also suggests a gap in our current understanding of Mtb biology. We attempt to bridge this gap by carrying out extensive re-annotation and constructing a systems level protein interaction map of Mtb with an objective of finding novel drug target candidates. Towards this, we synergized crowd sourcing and social networking methods through an initiative ‘Connect to Decode’ (C2D) to generate the first and largest manually curated interactome of Mtb termed ‘interactome pathway’ (IPW), encompassing a total of 1434 proteins connected through 2575 functional relationships. Interactions leading to gene regulation, signal transduction, metabolism, structural complex formation have been catalogued. In the process, we have functionally annotated 87% of the Mtb genome in context of gene products. We further combine IPW with STRING based network to report central proteins, which may be assessed as potential drug targets for development of drugs with least possible side effects. The fact that five of the 17 predicted drug targets are already experimentally validated either genetically or biochemically lends credence to our unique approach.

## Introduction

Proclaimed a global health emergency by the World Health Organization (WHO) in 1993, Tuberculosis (TB) still remains the leading cause of mortality and affects approximately 32% of the world population [1]. The emergence of multi-drug-resistant strains of *Mycobacterium tuberculosis*, the causative agent of TB, and the vulnerability of the patients infected with HIV to tuberculosis have not only fuelled the spread of the disease but also present a challenging task of understanding the disease physiology and discovering new drug targets. In this quest, Mtb was sequenced and annotated in 1998 [2]. A subsequent re-annotation in 2002 successfully assigned functions to almost half of the approximately 4000 genes [3]. More recently, 20 more ORFs have been added to this list and the annotations updated [4], [5]. However a huge gap in information exists between published literature and the genome databases. The existing annotations in these databases are thus insufficient to generate the protein interaction map or the interactome, pivotal to understanding Mtb biology and identification of novel drug targets. To this end, Open Source Drug Discovery (OSDD) project (www.osdd.net) [6], [7] launched the Connect to Decode (C2D) program (http://c2d.osdd.net), an innovative blend of crowd sourcing and social networking in a virtual cloud space for a comprehensive collaborative re-annotation of Mtb which is the primer for generating the interactome. The ultimate objective is to identify drug targets based on better understanding of the complex interactions of various biological macromolecules in the pathogen.

Systems biology-based approaches have been applied to obtain better insights into the pathogen biology [8]. This strategy may help in identifying more than one potential drug targets and these can be utilized as sets of targets for a polypharmacology approach. A promising candidate in this category is bi-substrate acyl-sulfamoyl analogues that simultaneously disrupt crucial nodes in biosynthetic network of virulent lipid with dramatic effect on the cell surface architecture of Mtb [9]. Also, a recent study on genome-wide siRNA experiment has identified host factors that regulate Mtb load in human macrophages and are crucial to understand the dynamic interplay of molecular components of the pathogen and the host [10]. There are many such studies that try to capture the snapshots of the molecular interactions in Mtb in different conditions. It is therefore imperative to capture and curate data on experimentally validated interactions lying scattered in diverse sources in the literature to generate a genome scale network. This was achieved through the C2D program. The C2D community started with initial registration of more than 800 researchers, which largely consisted of research scholars, graduate students and under-graduate students. The participants were trained, evaluated and filtered at various stages of online training and assignments (https://sites.google.com/a/osdd.net/c2d-01/pathwayannotationproject/results-of-the-exercise). More than 100 researchers were selected as curators to obtain the final annotations (https://sites.google.com/a/osdd.net/c2d-01/pathwayannotationproject).

Here we describe how C2D has implemented a community annotation approach in a distributed co-creation mode for mining literature and how the accuracies and scope of assigning functions were enhanced using combined evidence approach. We have enriched the annotations of the Mtb genome both in terms of coverage and details (**Table 1**). Web2.0 collaborative online tools enabled voluntary community participation for implementing this task. An important part of the project was creating self-organized communities to collectively learn and share the process and the standards for reporting annotations. As per published estimates, this innovative approach packed nearly 300 man-years into 4 months [11] and it has also established a novel way of collective problem solving on a voluntary basis in a sustainable manner [12]. This is, to the best of our knowledge, lead to the creation of the largest manually curated interactome of Mtb. Based on the varied nature of interactions among proteins in vivo, we propose a new network definition called “Protein-Protein Functional Network” (PPFN). This network encompasses a total of 1434 proteins connected through 2575 functional relationships. In this paper, we detail how the Interactome - PathWay (IPW), an open collaborative platform was used to generate and analyze potential drug targets. Using betweenness centrality [13] as a first indicator to shortlist candidate drug targets, we zeroed into 73 proteins. We have in the process also created a sustainable open innovation platform.

**Table 1:** The data structure that was used to capture the interactome data.

Field	Description
GeneID	The unique identifier for a given gene (Rv ID and NCBI Gene ID)
Gene Name	Assigned name of the gene
Pathway	Biological role of the gene
Gene function	The biological function of the gene
Interacting Partners	All the interacting partners for a given gene
Type of Interaction	Type of interaction (protein-protein [p-p], protein-nucleotide [p-n])
Nature of Interaction	This field contains nature of interaction, such as structural complex, regulatory, signaling etc.
Method of Inferring Interaction	Contains information about the experimental or computational methods used for the inference of interacting partners
Type of Evidence	Type of evidence, adopted from Gene Ontology (IDA, IPI, ISO, TAS, etc)
PUBMED/Link of source	PubMed ID or any web based link from where the interaction and other annotation details were inferred
Email of author	E-mail address of curator

There were 11 annotation fields for reporting annotations. The data is available in PSI MITAB format.

## Results and Discussion

### C2D Annotation

**Figure 1:**
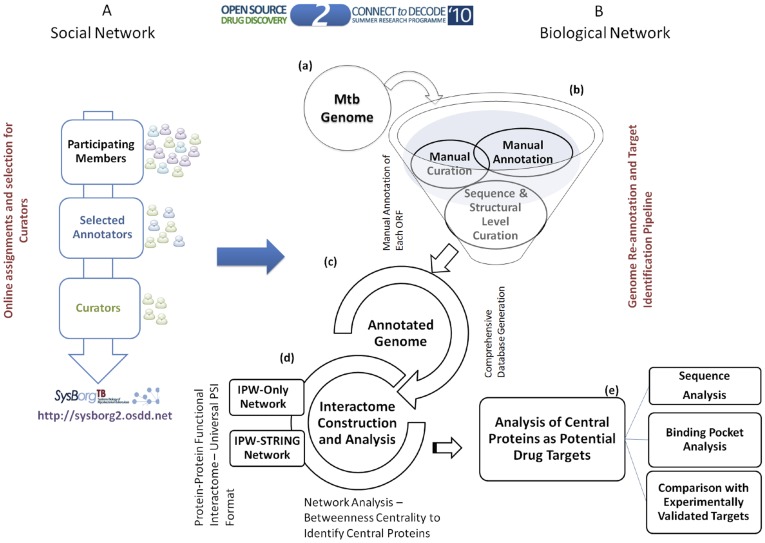
From Social Network to Biological Network. The C2D annotation approach for manual annotation and curation of Mtb interactome followed by network analysis to predict potential drug targets reported at various sequence and structural level filters. (A) Illustrates the overall approach of crowd sourcing through social network implemented in C2D exercise (B)(a) Mtb Genome (b) Manual collation and sequence/structure based curation for gene annotation (c) Collation of re-annotated genome into comprehensive data structure (d) Construction of protein-protein interaction network based on the annotated data (e) Target identification using network analysis; Sequence level comparison of selected proteins with that of human homologs, human gut flora and human oral flora; systems, sequence and structure level analysis of shortlisted proteins and experimentally validated drug targets.

An overview of the approach followed in *‘Connect to Decode’* (C2D) exercise is as illustrated in **Figure 1**. Broadly the approach was designed based on the principles of the fourth paradigm of science, encompassing data collation, curation and analysis [14]. Roughly ∼4.4 Mbp genome of Mtb was re-annotated manually. To streamline the annotation process and select a community of researchers competent to implement this project, a series of online assignments and training modules were assigned (see methods). These steps ensured the selection of serious and dedicated contributors thereby assuring the quality of data collation, curation and analysis. Various standard operating protocols (SOPs) were designed and shared with the participants for the consistency in the steps followed for the annotation of genes (https://sites.google.com/a/osdd.net/c2d-01/pathwayannotationproject/instructions-for-annotation and https://sites.google.com/a/osdd.net/c2d-01/pathwayannotationproject/example-annotation and https://sites.google.com/a/osdd.net/c2d-01/pathwayannotationproject/stepsforproteinannotation ). Given the exponential increase in the number of publications from about 300 per year since 1990’s to a staggering 2000 per year in 2010, the challenging task of collating and curating data was achieved through the formulation of community editable interactive platform designed to facilitate real time annotations and continuous updates. The community scanned and retrieved information from nearly 10,000 published studies in addition to extracting information from databases and transferred annotations using sequence and structure analyses based approaches. The community has cited more than 3000 papers in annotation process as on an average 3–4 manuscripts were referred or read in order to get the relevant information to annotate a given protein.

### The Mtb Genome Annotation and Interactome Curation

**Figure 2:**
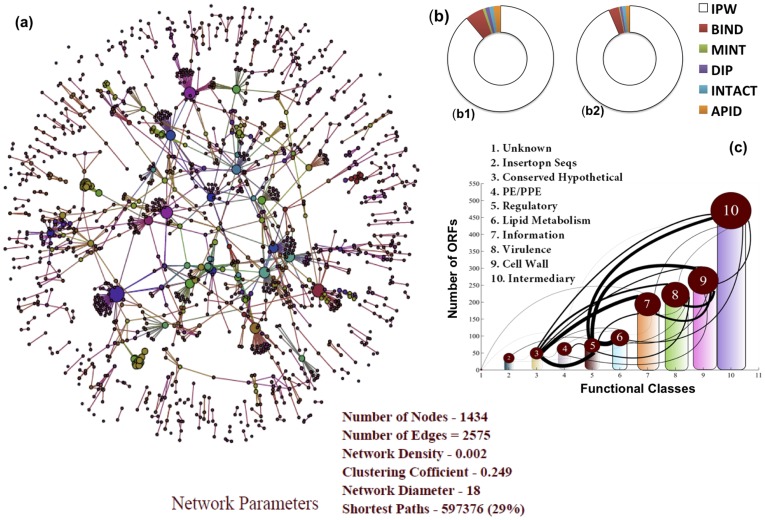
IPW interactome and comparison with existing annotation databases (a) IPW-Only protein-protein functional interaction network, (b) Comparative analysis of IPW-Only proteins and interaction with existing manually curated databases, Ring represents all interactions and proteins in IPW displaying the subsets which are obtained from other manually curated databases (b1) Comparative analysis of IPW-Only interactions to that of existing manually curated databases (b2) comparative analysis of protein as curated in IPW-Only to that of proteins presents in other manually curated databases (c) TubercuList functional class interaction relation based on the interactions as obtained from IPW-Only. The connectivity (lines) represents the interacting proteins within these classes.

IPW has resulted in annotation of 87% of the genome in the context of reporting gene products as compared to 52% in the re-annotation reported in 2002. Moreover, less than 5% of the interactions in IPW (Table S1) exist in other manually curated interaction databases such as BIND [15], APID [16], IntAct [17], DIP [18] and MINT [19] (**Figure 2(b)** **)**. Thus**,** to the best of our knowledge, *Connect to Decode’s* Interactome Pathway Annotation (IPW) has generated the largest data set of manually curated interactions in Mtb. These interactions not only include data from large interaction databases such as IntAct, BIND, MINT, APID, DIP, etc but also include a large amount of manually curated information from literature.

Of the 1193 hypothetical proteins from TubercuList [4], the IPW based annotations identify gene products for 770 proteins. Of the 1480 hypothetical proteins reported in KEGG [20] database, functional associations have been made to 1055 proteins, clearly showing how IPW has bridged the wide gap that existed between information captured in databases and that available in literature. To ensure that IPW remains up to date, the data from IPW is shared with members of the OSDD community in an ‘edit’ mode, through which new interactions can be added using the SOP that includes a rigorous quality check phase, specifically designed for community contribution.

### Interactome Construction: IPW and Combined Network with STRING

Interactome as a whole constitutes various biological interactions belonging to both structural and functional type of protein-protein associations. To have an encyclopedic view of various interactions that take place at protein functional level, we report the construction of two types of networks. The first network, termed IPW only (**Figure 2(a)** **)**, was constructed on the basis of the IPW curated data alone. The nodes in the network represent the proteins whereas the edges represent the functional interactions among those proteins. The nodes were scaled and color coded in proportion to their degrees. Also, based on the common interactions we derived a connectivity relationship between various TubercuList functional classes [4]. **Figure 2(c)** shows the connectivity among 10 broad functional classes of TubercuList. The edge thickness was taken to be directly proportional to the number of common proteins between the two TubercuList functional classes for the given pair. Significant functional dependencies are seen among the ‘Lipid Metabolism, Cell Wall, Intermediary metabolism and Regulatory systems’ functional classes, reflected in their edge thicknesses in the network. Disruption of such linkages can lead to breakdown of crosstalk between these biological processes and thus could be exploited to identify new drug targets.

Secondly, in order to obtain insights on the complete functional organization among all the possible proteins of Mtb, a combined network termed, IPW-STRING (IPWSI), was constructed by overlaying STRING network on the IPW network. The STRING based network of Mtb was derived from STRING 8.0 [21] database consisting of various interactions among proteins as derived on the basis of extensive computational and limited experimentally inferred interactions. Computational predictions have been based on established methods such as phylogenetic profiling, domain fusion, common gene neighborhood and operon criteria. However, computational models over predicts interactions since they do not account for spatio-temporal separation of the interacting partners. Thus, in the combined network the accuracy of interaction decreases whereas the coverage increases. It should also be noted that there is an inherent bias for well-studied proteins in IPW. A simple comparison shows that nearly 60% of IPW interactions have experimental evidence codes as compared to 2% existing in STRING. Also, about 29 additional proteins and 1762 new functional interactions apart from that reported by STRING were included in the new IPW-STRING combined interactome.

The combined IPW-STRING interactome was further used to decipher various possible drug targets using the concepts of graph theory. The network analysis of these networks provides a means to understand the functional organization of the organism from the network topology point of view [22], [23]. Various network properties as computed for both the networks and their biological relevance are discussed below.

### Topological Organization of Interactome

In order to understand the functional organization of constructed interactome we further assessed the fundamental properties of this network from the graph theoretic point of view. Given a vast interaction space encompassing the interactome as whole, where the nodes represents proteins and interaction represents a functional relation between them, it becomes imperative to understand the functional organization of the network from its topology. The most fundamental characteristic of a graph is the connectivity of its constituent nodes as represented by the degree. Degree, being a measure of interconnectedness of nodes highlight the importance of a node (protein in this case) with respected to other nodes in the network. A maximum degree of 44 and 289 was observed for the IPW and IPWSI networks, respectively, suggesting the level of maximum number of functional relation of a given protein in both the networks.

**Figure 3:**
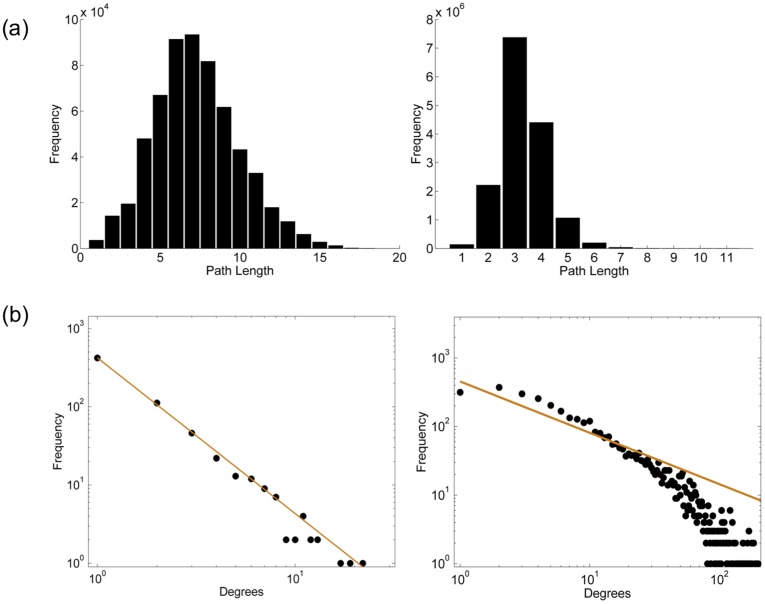
Network parameters (a) Characteristic path length of IPW-Only network and IPWSI network. In both the graphs the x-axis represents the path length whereas the y-axis represents the frequency. 3(b) Log-Log plot of degree distribution of IPW network, the solid line was obtained by fitting the power law for γ  = 1.99 and Log-Log plot of degree distribution of IPWSI network, the solid line represents the power law fit with γ  = 2.01.

Clustering coefficient for a node indicates the connectivity of the neighbours of a given node to the other nodes in the network [24]. This parameter was computed to elucidate the dependencies of two or more proteins with respect to each other and to rest of the proteins in the network. The clustering coefficient for IPW and IPWSI networks was observed to be 0.249 and 0.377, respectively. The high clustering coefficient of both the networks suggests the presence of well-connected hubs within the network, which are important from the functional crosstalk between the proteins of Mtb. Further, the characteristic path length of both the networks was computed in order to comprehend the extent of functional relation between any two given proteins in the network. The characteristic path length of both the networks is as shown in **Figure 3(a)**. The characteristic path length in IPW network was observed to be 7.2 whereas for IPWSI it was observed to be 3.13. From the network navigability point of view the characteristic path length can be inferred as the number of steps that one has to take traversing from one node to other, which from biological point of view could be inferred as the amount of communication that is possible between any two proteins. Pertaining to the high characteristic path length of IPW alone, the absence of functional relation between any two proteins can be inferred; however, the functional relation between any two proteins increase when the IPW alone was clubbed with STRING based network. The characteristic path length, thus, can be used to understand the functional gap that possibly exists in the protein-protein interaction network. Emphasizing on the network communication further, the network diameter was computed representing the length of the ‘longest’ shortest path in the network. The network diameter of IPW and IPWSI networks was observed to be 18 and 10, respectively. Akin to characteristic path length, the network diameter can be used to interpret the overall navigability of the network, the higher the diameter, the more distantly two nodes are related and *vice versa*.

As discussed, understanding the topological organization of the network could lead to better understanding of its underlying principles. The network topology could also be used to understand the number of possible modules (hubs) in the network, which may help in identifying potential drug targets. In order to obtain such insights, we tested the existence of power law distribution on IPW and IPWSI networks, respectively. The power law distribution can also be used to understand the scale free nature of a network [23]. There is extensive literature that reports the existence of scale free nature of biological networks. The power law distribution on the node degree distribution of IPW and IPWSI networks is shown in **Figure 3(b)**. The value of γ was observed to be 1.99 for IPW and 2.01 for IPW-STRING combined node degree distribution.

### Target Identification

Apart from inferring fundamental principles of network properties the availability of an interactome also enables prediction of essential proteins from the network structure point of view. The protein lethality within a network is usually obtained from the degree distribution of the nodes in the networks. The nodes with high degree are considered important and hence regarded as probable drug targets. The degree distribution alone could lead to improper putative drug target identification as it does not capture the alternate routes in the network. Most of the biological networks possess large number of shortest paths [25]. The large number of shortest paths also suggests the availability of alternate routes within the network which could be used to achieve a certain biological objective. Removing such nodes from the network could lead to maximum disruption in the network. In order to capture these properties, important nodes as well as important edges, we used betweenness centrality [24], [26] as a metric system to infer putative drug targets. The node betweenness centrality at a threshold of ≥0.2 lead to identification of 17 and 64 central proteins from IPW and IPWSI networks, respectively (Table S2).

### Analysis of Putative Drug Targets: Identifying Probable Non-toxic Targets

To design a viable drug it is essential to ensure least probability of off-target interactions. A sequence, structure and systems based analysis was performed in order to predict the druggability of the shortlisted central proteins from the two networks so as to reduce the chances of off-target interactions.

**Figure 4:**
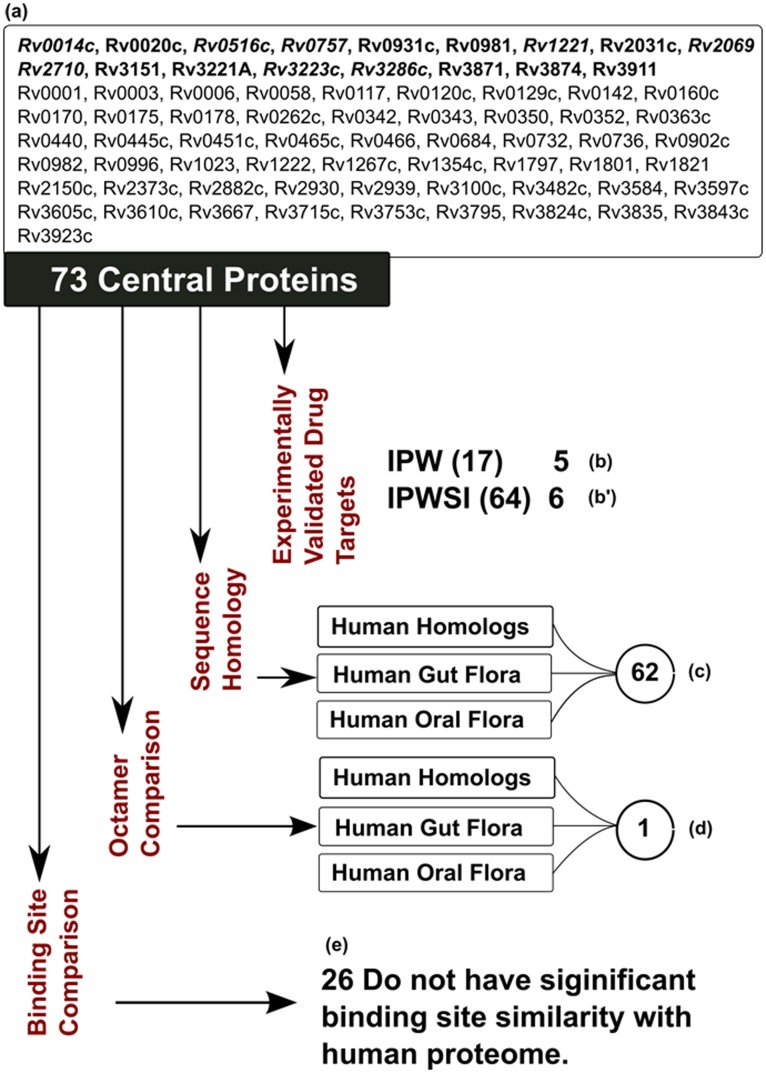
Illustrates the comprehensive analyses of central proteins as potential drug targets. The various filters include comparison with validated drug targets, sequence and structural level comparison with Human proteome, gut and oral flora (a) The list of 73 central ORFs wherein Rv Ids in bold represent IPW central ORFs, Rv IDs in regular font represents IPWSI central ORFs and the italicized-bold represent common Rv Ids to both IPW and IPWSI. (b & b’) Five of the 17 IPW and six of 64 central ORFs with experimental validation as drug targets. (c) Sequence homology comparison with human proteome and human microbiome results in 62 ORFs with no significant similarity (d) Octamer analyses against human proteome and human microbiome results in one ORF with no hits (e) Comparative binding site analysis with human proteome results in 26 ORFs with no significant similarity (lists b, b’, c, d and e available in Table S2).

The list of 17 and 64 proteins (73 unique proteins as eight are common in the two lists) was first filtered against human homologs and human oral and gut flora [27]. Of the 17 targets identified by IPW, none had a homolog in human proteome and in human oral and gut flora. In the combined network IPWSI, 53 such targets were identified (**Figure 4** **)**. There are 62 unique central proteins without any significant homology to human proteome, gut and oral flora from IPW and IPWSI. We further analyzed this list of 62 proteins for absence of small peptides (octamer) since it has been reported that a small fraction of peptide sequences are evolutionarily conserved and invariant across several organisms [28]. These peptide sequences can adopt similar conformation in different protein structures [28]. A comparative analysis shows that one protein Rv3221A does not share any common octapeptide with human proteome, gut or oral flora. However, a closer and detailed analysis needs to be performed for proteins sharing octapeptide with human proteome and human microbiome in order to evaluate their status for off-target binding. In order to understand the binding pockets, an independent analysis has been performed to predict and match binding pockets of central proteins with human proteome. Of the 73 central proteins, 57 have either PDB or ModBase [29] structure making them amenable to structural analysis for druggability. We analyzed these proteins as per the targetTB [30] pipeline where the top 10 binding sites for each of these 57 proteins were identified using PocketDepth algorithm [31]. The binding pockets of these proteins were then compared with human proteome using PocketMatch [32]. Of the 57 proteins, 31 proteins have high structural similarity with human proteome at binding site level whereas 26 proteins which do not have binding site similarity with human proteome. It is interesting to note that seven of these are experimentally validated drug targets.

**Figure 5:**
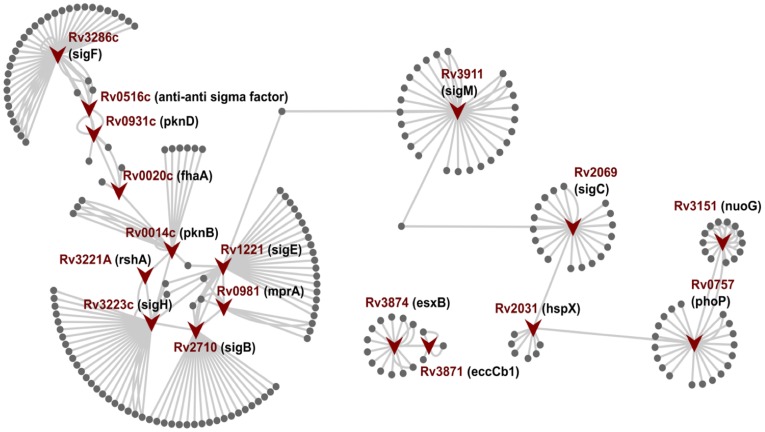
Illustration of 17 putative drug target interaction from IPW interactome depicting the cascade of how the central proteins interact with each other in a spatio-temporal manner under different conditions like growth, stress and survival in macrophages including virulence. Under normal conditions, PknB phosphorylates RshA which inhibits SigH. However, under oxidative stress, RshA is not phosphorylated and this abolishes its binding to SigH, rendering it free. SigH in turn upregulates expression of SigE and SigB which regulates MprA (bacterial persistence regulator). MprA also regulates SigB and SigE. SigB plays important role in adaptation to stationary phase and nutritionally poor conditions and SigE is upregulated in mycobacterial growth within human macrophages and is transcribed from three different promoters under different conditions. sigB is also regulated by SigF, which regulates the expression of genes involved in the biosynthesis and structure of the mycobacterial cell envelope, including complex polysaccharides and lipids, particularly virulence- related sulfolipids and several transcription factors. Rv0516c is an anti-anti sigma factor and regulates anti-sigma factor SigF (upregulated during infection culture of human macrophages and in nutrient starvation condition; regulates transcription of genes involved in cell wall biosynthesis, sulfolipid metabolism, nucleotide metabolism, energy metabolism and several transcription factors) on getting phosphorylated by PknD which in turn is regulated by Rv0020c phosphorylated by PknB and PknE. SigF also regulates sigC and regulates hspx that is also regulated by dosR regulon. dosR regulon in turn is again regulated by PhoP which is a transcription factor for nuoG, eccCb1, esxb/cfp10 [48].

Rv3221A (RshA) (**Figure 4** **List d**), an anti-sigma factor to the primary stress response sigma factor SigH, passed all filters but is neither reported as a potential drug target in literature nor in targetTB predictions. The gene encoding RshA lies in the same operon as SigH and is co-expressed with the same [33]. It has a strong affinity to bind with SigH and attenuates its ability to bind to the RNA polymerase holoenzyme under normal growth conditions. Under conditions of oxidative stress, phosphorylation of RshA by Rv0014c (PknB) abolishes its binding to SigH, which in turn leads to the cascade of expression of several stress response proteins [34] (**Figure 5**). SigH causes increased expression of two other sigma factors Rv2710 (SigB) and Rv1221 (SigE), which are also known to be stress related sigma factors that assist Mtb in its survival during several stress conditions and are also central proteins. The other interacting partners of RshA include heat shock proteins and chaperones like Rv0384c (ClpB) and Rv0350 (DnaK), enzymes for oxidative stress response Rv1471 (TrxB1), Rv3913 (TrxB2) and Rv3914 (TrxC) which are also part of the sigH regulon. sigH also induces enzymes involved in cysteine biosynthesis and in the metabolism of ribose and glucose for the production of mycothiol precursors, which assist in cellular protection under oxidative stress. The SigB and SigE signaling cascade downstream interacts and regulate other central proteins (**Figure 5**). RshA is at the beginning of this cascade and seems to play in crucial role in regulating the stress response proteins, starting with sigH.

The objective of the interactome construction and analyses was to identify central proteins, which have significant roles in maintaining growth and survival of the bacterial pathogen. We identified 17 such central proteins (**Table 2**), five of which (*PknB*, *NuoG*, *PhoP*, *EccCb1*, *HspX*) have been previously functionally characterized and shown to be essential by gene deletion and mutation and thus are considered as validated drug targets. The target gets further validated if there are inhibitors which inhibit the function of the target enzyme or protein as well. *PknB* (Rv0014c) is an essential serine-threonine protein kinase in Mtb and has role in a number of signaling pathways in cell division and metabolism. Several inhibitors have been reported for this kinase [35] and is also one of the targets being pursued by Working Group on New TB Drugs (http://www.newtbdrugs.org/project.php?id=81). *NuoG* (Rv3151) is a subunit of type I NADH dehydrogenase playing an important role in growth in macrophage and pathogenesis in animal models [36]. *PhoP* (Rv0757), a response regulator and part of the two component system, when mutated leads to growth defects in macrophages and in mouse models [37]. *eccCb1* (Rv3871) is a part of the RD1 region and mutation leads to attenuated growth and toxicity in THP-1 cells. The mutants cannot export CFP-10 and are avirulent [38]. *hspX* (Rv2031c) encodes for a alpha-crystallin-like protein and plays a significant role in retaining a non-replicating state in latency [39], [40]. The fact that five of the 17 putative drug targets from IPW are already validated drug targets, lends credence to our approach of annotating the genome and interactome construction of Mtb for system level understanding towards novel drug target identification.

**Table 2:** The list of all the 17 central proteins as predicted from the betweenness centrality of >0.2 through IPW network with their gene products.

Accession	Gene Name	Description (Gene Product)
Rv0014c* ^[**35**]^	pknB	TRANSMEMBRANE SERINE/THREONINE-PROTEIN KINASE B PKNB (PROTEIN KINASE B)
**Rv0020c**	fhaA	CONSERVED HYPOTHETICAL PROTEIN WITH FHA DOMAIN, TB39.8
**Rv0516c**	Rv0516c	ANTI-ANTI SIGMA FACTOR
Rv0757* ^[**37**]^	phoP	POSSIBLE TWO COMPONENT SYSTEM RESPONSE TRANSCRIPTIONAL POSITIVE REGULATOR
**Rv0931c**	pknD	TRANSMEMBRANE SERINE/THREONINE-PROTEIN KINASE D PKND (PROTEIN KINASE D)
**Rv0981**	mprA	MYCOBACTERIAL PERSISTENCE REGULATOR MRPA (TWO COMPONENT RESPONSE TRANSCRIPTIONAL REGULATORY PROTEIN)
**Rv1221**	sigE	ALTERNATIVE RNA POLYMERASE SIGMA FACTOR SIGE
Rv2031c* ^**[39]**, [**40**]^	hspX	HEAT SHOCK PROTEIN HSPX (ALPHA-CRSTALLIN HOMOLOG) (14 kDa ANTIGEN) (HSP16.3)
**Rv2069**	sigC	PROBABLE RNA POLYMERASE SIGMA FACTOR, ECF SUBFAMILY
**Rv2710**	sigB	RNA POLYMERASE SIGMA FACTOR
Rv3151* ^**[36]**^	nuoG	PROBABLE NADH DEHYDROGENASE I (CHAIN G) NUOG (NADH-UBIQUINONE OXIDOREDUCTASE CHAIN G)
**Rv3221A**	Rv3221A	POSSIBLE ANTI-SIGMA FACTOR RSHA
**Rv3223c**	sigH	ALTERNATIVE RNA POLYMERASE SIGMA-E FACTOR (SIGMA-24)
**Rv3286c**	sigF	ALTERNATE RNA POLYMERASE SIGMA FACTOR
Rv3871* ^**[38]**^	Rv3871	ESX CONSERVED COMPONENT ECCCB1 (ATPase activity)
**Rv3874**	esxB	10 KDA CULTURE FILTRATE ANTIGEN ESXB (LHP) (CFP10)
**Rv3911**	sigM	RNA POLYMERASE SIGMA FACTOR

RvIDs superscripted with asterisk are essential proteins as evidenced by genetic and biochemical studies.

Despite the efforts over a number of years, discovering novel, fast acting drugs for TB has been a major challenge. However, recently introduced combination drug Risorine designed on the principles of Ayurveda has been shown to cut down rifampicin use leading to very high compliance [41]. Understanding the biology of the pathogen through a systems level approach can help in identifying the Achilles heel for Mtb. Towards this, Interactome Pathway annotation has captured the updated relevant information on Mtb genes and has tried to unravel the puzzle. We have amalgamated crowd sourcing with social networking to comprehensively reannotate the Mtb genome, generated its largest ever interactome and propose potentially efficacious drug targets. In the process, we have set up an open collaborative platform and a dynamic community to ensure regular updates.

## Materials and Methods

### Crowd Sourcing for Data Curation

#### Data capture

Two annotation standard operating protocols (SOPs), in the presence of literature and in the absence of literature, were designed in order to capture the maximum amount of relevant data. Wherever the protein was not studied in Mtb, the annotations were transferred from other organisms based on conservative statistical measures in sequence and structure-based analysis as discussed below (i and ii). To ensure consistency and integrity of the data added to the resource, Standard Operating Protocols (SOPs) were created and followed by the community. These SOPs and tutorials may be accessed at (http://c2d.osdd.net and https://sites.google.com/a/osdd.net/c2d-01/pathwayannotationproject).

#### Annotation SOP in presence of literature

The first step was to retrieve information on Mtb proteins with experimental evidence from literature. PubMed and Google based literature searches were carried out using suitable keywords, such as the respective Rv number, gene name, *Mycobacterium tuberculosis*, along with suitable Boolean expressions, such as AND and OR (for example, [Rv1018c] AND [mycobacterium tuberculosis], [epoxide hydrolase] AND [mycobacterium tuberculosis]). While manually scanning the available literature, emphasis was placed on the references, which dealt with *Mycobacterium tuberculosis* H37Rv followed, by evidence in other mycobacterial species. If the protein was an enzyme, the corresponding reaction, along with the EC number, and the pathway(s) in which the protein participates was also recorded.

#### SOP for annotation in the absence of direct information from literature

In absence of direct literature information, annotations were derived based on sequence, structure and profile based information and analyses. To begin with, NCBI-BLAST [42] was used to obtain homology information of the query protein. Hits with e-value of ≤0.0001 and identity of ≥35%, with ≥75% sequence coverage were considered as significant hits. Annotations of the closest homologue were transferred and recorded in the template against each annotation. Similarity search in the Pfam [43] database was carried out to support BLAST results and also to annotate in the absence of high query coverage with BLAST analysis. If both BLAST and Pfam similarity search failed to give a significant hit, Phyre [44], an automatic fold recognition tool was used for predicting the function of the Mtb proteins through high confidence fold associations. Appropriate evidence codes have been used to distinguish between transferred annotations and experimental based annotations.

#### Data curation

Multiple rounds of collaborative data quality checks were carried out to ensure that the data has been correctly captured and reported. A set of instructions (SOPs) were devised for the same (https://sites.google.com/a/osdd.net/c2d-01/pathwayannotationproject/data-qc-guide) where the annotations curated by the members were systematically crosschecked iteratively by other members. It was interesting to note that high quality curation was achieved by this approach of ‘many eyeballs make the bug shallow’, a common phenomenon in open source software projects.

#### Data organization

The collated data was organized into a defined data structure as depicted in **Table 1** with two columns, field and description. The PSI MI (Proteomics Standards Initiative Molecular Interactions) was used as the community standard for reporting protein-protein interactions in MITAB format (Table S3). This helps in improving the representation of molecular interaction data and its accessibility to the user community.

### Interactome Construction and Network Parameter Estimation

#### IPW and IPWSI network

The IPW-only network was constructed based on the annotations and curation of the data from IPW. The combined network of IPW and STRING termed, IPWSI, was constructed by combining the IPW network with that from STRING. All the interactions in STRING with high and medium level confidence score (above 400) were used to construct STRING based protein-protein interaction network. Methods used to compute network parameters are discussed below.

#### Network properties

To understand the functional organization of interacting proteins in both the networks, an analysis of various network topologies was performed. These network properties were computed using Boost Graph library in MATLAB (David Gleich; http://www.stanford.edu/~dgleich/programs/matlab_bgl/).

#### Connectivity or degree

The most elementary characteristic of a node in the network is its degree k, which represents, for a given node the number of other nodes it is connected to.

#### Clustering coefficient

The clustering coefficient was first defined by Watts and Strogatz [24]. The clustering coefficient, *C*, for a node is a notion of how connected the neighbours of a given node are to the other nodes (*cliquishness*) [45]. The average clustering coefficient for all nodes in a network is taken to be the network clustering coefficient. In an undirected graph, if a vertex *vi* has *ki* neighbors, *k i* (*k i* - 1)*/*2 edges could exist among the vertices within the neighbourhood (*Ni* ). The clustering coefficient for an undirected graph **G**(**V**, **E**) (where **V** represents the set of vertices in the graph **G** and **E** represents the set of edges) can then be defined as
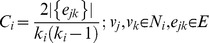



The average clustering coefficient characterizes the overall tendency of nodes to form clusters or groups. *C*(*k*) is defined as the average clustering coefficient for all nodes with *k* links.

#### Characteristic path length

The characteristic path length, *L*, is defined as the number of edges in the shortest path between two vertices, averaged over all pairs of vertices. It measures the typical separation between two vertices in the network. Intuitively, it represents the network’s overall navigability [45].

#### Network diameter

The network diameter *d* is the greatest distance (shortest path, or geodesic path) between any two nodes in a network. It can also be viewed as the length of the ‘longest’ *shortest path* in the network.
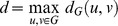
where d**_G_** (*u, v*) is the shortest path between *u* and *v* in **G** [45].

#### Power law distribution

For a given network the power law distribution states the probability that a given node has k links, which is given by equation p(k) ∼ k-γ, where γ is degree exponent. For smaller values of γ, the role of the ‘hubs’, or highly connected nodes, in the network becomes more important. For γ >3, hubs are not relevant, while for 2<γ <3, there is a hierarchy of hubs, with the most connected hub being in contact with a small fraction of all nodes. Scale-free networks have a high degree of robustness against random node failures, although they are sensitive to the failure of hubs [23]. The probability that a node is highly connected is statistically more significant than in a random graph [45].

#### Betweenness centrality

The betweenness centrality is the measure of vertex within a graph. For a given graph G(V,E), with n vertices, the betweenness CB (v) of a vertex v is defined as.
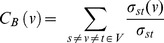
where σ_st_ is the number of shortest path from s to t, and σ_st_ (v) is the number of shortest paths from s to t that passes from vertex v. The betweenness centrality analysis was performed for both the networks [45]–[46].

### Drug Target Identification

#### Sequence homology with human proteome, oral and gut flora

The complete human proteome was downloaded from NCBI and BLAST was used to filter out the proteins, which had homology of greater than 45% with human protein. Human gut and oral flora constitutes the microbes that are considered to influence the physiology, nutrition, immunity and development of host. The complete proteome of 8-gut flora and 27 oral floras were downloaded. CD-HIT with similarity of 60% and a word size of 4 was used to compare the predicted proteins [27].

#### Binding site similarity with human proteome, oral and gut flora

We analyzed these proteins as reported in targetTB [30] pipeline where the top 10 binding sites for each protein was identified using PocketDepth algorithm [31]. The binding pockets of these proteins were then compared with human proteome using PocketMatch [32].

#### Peptide level conformation comparison with human proteome, oral and gut flora

We analyzed the proteins for absence of small peptides (octamer) [28] across human proteome, gut or oral flora using in house PERL scripts.

#### Literature based target validation

The predicted targets were further validated based on presence of existing functional evidence in literature. Data-mining and manual curation was performed to identify and document validated drug targets in Mtb. In addition to this, it was also documented whether the central protein is also reported to be essential or non-essential in context of Mtb growth and survival.

### Web Server for Accessing and Searching IPW

The IPW data has been posted on http://sysborg2.osdd.net, the semantic web-based platform of Open Source Drug Discovery (OSDD) project [47]. For ease of access and search, the data is provided through a web-based server available at http://crdd.osdd.net/servers/ipw built using PHP and Mysql. This also works as the annotation and curation interface for the community. Any new submission to this web servers requires http://sysborg2.osdd.net open ID for authentication so that appropriate credits may be given to the members submitting updated information.

## Supporting Information

Table S1 **The annotations in the data structure format described in** **Table 1** **.** This data may be searched in customized manner using the IPW web-interface (http://crdd.osdd.net/servers/ipw).
(XLSX)

Table S2 **Rv Ids in lists b, b’, c, d and e as obtained from various sequence and structural level analysis of central proteins as potential drug targets from IPW and IPWSI as depicted in** **Figure 2** **.**
(XLSX)

Table S3 **Central proteins predicted from analysis of the IPW interactome with details of interacting partners in PSI MITAB format.**
(XLSX)
